# Evaluating the national system for rare diseases in China from the point of drug access: progress and challenges

**DOI:** 10.1186/s13023-022-02507-2

**Published:** 2022-09-10

**Authors:** Luyao Qiao, Xin Liu, Junmei Shang, Wei Zuo, Tingting Xu, Jinghan Qu, Jiandong Jiang, Bo Zhang, Shuyang Zhang

**Affiliations:** 1 Department of Pharmacy and State Key Laboratory of Complex Severe and Rare Diseases, Peking Union Medical College Hospital, Peking Union Medical College, Chinese Academy of Medical Sciences, Dongdan Campus No. 1 Shuaifuyuan Wangfujing Dongcheng District, Beijing, 100730 China; 2 State Key Laboratory of Bioactive Substance and Function of Natural Medicines, Institute of Materia Medica, Peking Union Medical College, Chinese Academy of Medical Sciences, Beijing, China; 3 Department of Cardiology and State Key Laboratory of Complex Severe and Rare Diseases, Peking Union Medical College Hospital, Chinese Academy of Medical Sciences, Dongdan Campus, No. 1 Shuaifuyuan Wangfujing Dongcheng District, Beijing, 100730 China

**Keywords:** China’s national system for rare diseases, Orphan drugs, Accessibility, Availability, Affordability

## Abstract

**Background:**

There are about 7000 rare diseases worldwide, of which only 5% of the diseases can be treated with medicines, showing that it’s important to improve patient access to orphan drugs. Recently, China has actively worked to set up a national system for rare diseases to improve the diagnosis and treatment capabilities and ensure the accessibility of drugs. However, the benefits of the system have yet not to be measured. This study aimed to provide an overview of orphan drug access based on the Compendium of China’s First List of Rare Diseases and National Network to Collaborate on Diagnosis and Treatment of Rare Diseases, expecting to map a blueprint for orphan drug access in China.

**Methods:**

Framework of China’s national system for rare diseases was summarized. We surveyed the availability and affordability of 79 approved orphan drugs based on the Compendium of China’s First List of Rare Diseases in 30 leading provincial institutions from 2017 to 2020. The availability was measured annually at 3 levels (market, hospital and drug), and affordability was reflected by comparing costs of daily defined dose with per capita income of urban and rural residents, with the National Basic Medical Insurance considered.

**Results:**

The market availability of orphan drugs in China showed an upward trend. As of 2020, the median hospital-level availability was 41.1% (increased by 1.5 times), highly available drugs increased by 16.5%. There were 64/74 orphan drugs that were affordable to rural/urban residents with the National Basic Medical Insurance considered (an increase of 14.1%), and the urban–rural gap of affordability ratio was narrowed (down by 6.0%). Comprehensive analysis showed the proportions of drugs with better availability and affordability in urban and rural areas by 2020 were 39.4% and 32.3%, respectively, which had increased but were still at a low level.

**Conclusions:**

China’s national system for rare diseases has made great progress in orphan drug access, indicating that it’s been functioning under the joint reformation of medical treatment, medical insurance and medicines supply. The list of rare diseases will be updated and collaboration in networks will be enhanced to further improve the system.

**Supplementary Information:**

The online version contains supplementary material available at 10.1186/s13023-022-02507-2.

## Background

Rare diseases are diseases that affect a small number of the population. Although every single rare disease affects only an extremely limited number of patients, which is defined as affects no more than 1 person in 2000 in Europe and affects less than 200,000 people in the United States. There are a total of 7000 rare diseases along with 250 to 280 new additional ones annually [1, 2]. Rare diseases impact more people than cancer and AIDs, and approximately 30% of patients with rare diseases die before the age of 5 [3]. Although rare diseases vary in etiology and clinical manifestations, most of them are associated with significant disease burden [4, 5].

Orphan drugs are intended to treat, prevent or diagnose rare diseases. It was estimated that 95% of rare diseases are lacking drug treatments, demonstrating that the accessibility of orphan drugs is critical [3]. In addition, the high price of orphan drugs poses challenges for both patients and governments. For example, the annual cost of eculizumab is over $409,500, which is used to treat Paroxysmal nocturnal haemoglobinuria, and the annual cost of idursulfase is over $375,000, which is used to treat Mucopolysaccharidosis II [6]. Affordability of orphan drugs is a serious public health issue in China, as they are in other countries. A survey made by the Chinese Organization for Rare Disorders with 5810 patients registered in 2019 showed that the employment rate for adult patients was only 40%, and 80% of the income is spent on disease management. Poverty caused by the significant disease burden is a common experience for them and their families [7]. Orphan drugs, which account for a large proportion of treatment spending, deserves more attention.

The Orphan Drug Act was enacted in 1983 in the US. Only 10 orphan drugs had been approved by the US Food and Drug Administration (FDA) in the decade before 1983, while more than 350 orphan drugs had been approved by 2010. The Orphan Drug Act also inspired similar policies in Singapore, Australia, Japan and Europe [8]. China's attention to rare diseases started late. However, in recent years, the importance of orphan drugs has gradually appeared in various policy documents. For example, the Compendium of China’s First List of Rare Diseases (2018) (CLRD), which includes 121 rare diseases [9], first clarified the concept and scope of rare diseases in China. Besides, the CLRD has made China the first country to delineate the boundaries of rare diseases in the form of a catalogue. The National Network to Collaborate on Diagnosis and Treatment of Rare Diseases (NCDTRD) was established in China in 2019, consisting of 324 hospitals nationwide, including 1 leading national institution (Peking Union Medical College Hospital), and 32 leading provincial institutions, achieving rare disease resource sharing [10]. As the core contents of the national system for rare diseases in China, they played a key role in solving problems of rare diseases.

Improving orphan drugs access is a core priority for China’s policies. There have been studies assessing access to orphan drugs in Europe, the U.S., and South Africa [11–14]. However, very few studies have assessed the availability and affordability of orphan drugs in China, and most of them were made before release of the CLRD and NCDTRD [15, 16]. In comparison, for the first time on the basis of CLRD and NCDTRD, we evaluated the accessibility of orphan drugs that have been approved for CLRD indication in the U.S. [17], EU [18], Japan [19] and marketed in China in 30 leading provincial institutions of NCDTRD, exploring the concrete effects of the China’s national system from the point of drug access.

## Methods

### Data sources

Two forms of information were collected in this study. Refer to the disease types in CLRD, we confirmed orphan drug approvals and indication registration information from the FDA [20], European Medicines Agency [21] and Japan Ministry of Health, Labour and Welfare [22]. According to that, we then used the drug approval database from the National Medical Products Administration [23] to confirmed their marketing authorization. Different daily defined dose (DDD) information for rare diseases defined by WHO [24], which refers to the average daily doses of drugs prescribed for their major indications in adults, were used in this survey. There are five orphan drugs defined as DDD by WHO, including riluzole 100 mg, nusinersen 0.1 mg, macitentan 10 mg, bosentan 250 mg and miglustat 0.3 mg. However, for these drugs whose DDD information were not provided by WHO, packet inserts were the mainly way for us to obtain the dose information. A maintenance dosage regimen for moderate severity recommended in the packet inserts was used.

We also developed a survey questionnaire to collect the price and availability information of surveyed orphan drugs in 30 leading provincial institutions based on the methodology of the World Health Organization (WHO)/Health Action International (HAI) (2008) [25] (Additional file 1: Table S1. The questionnaire on the availability and prices of surveyed orphan drugs in leading provincial institutions).

### Selection of diseases and medications

There are 121 rare diseases included in the CLRD [9]. We included orphan drugs that have been approved for clear indications in the U.S./EU/Japan and have been marketed in China, and drugs without an approved indication were not included. There were 79 drugs for rare diseases were adopted in our survey (Additional file 3: Table S3 The flow chart for orphan drug screening in the survey). Further, the anatomical therapeutic chemical (ATC) classification system was used to identify the ATC code for each drug (Additional file 2: Table S2 shows the list of 79 orphan drugs surveyed in 30 public tertiary hospitals in China (99 drug-indication matches)) [26]. These drugs were divided into seven efficacy groups according to International Classification of Diseases 11th Revision (ICD-11) [27] (Additional file 4: Table S4 ICD-11 categories of surveyed drugs in China).

### Survey and selection of medical facilities

We surveyed the availability and prices of 79 orphan drugs in 30 leading provincial institutions of NCDTRD. The leading provincial institutions are selected based on the evaluation of the diagnosis and treatment capacity of rare diseases in each province. Therefore, we believe that our data can reflect the status of orphan drug access in China. Considering the economic disparities China, 30 institutions from 29 provinces were classified into 3 areas: eastern, middle and western. (Additional file 5: Table S5 shows the list of the surveyed 30 leading provincial institutions of the "National Network to Collaborate on Diagnosis and Treatment of Rare Diseases" in China.)

### Measures and analysis

Based on the analytical framework of WHO/HAI methodology, we measured accessibility of orphan drugs from availability and affordability with economic disparities considered.

#### Availability

Orphan drug availability in China was measured annually at market level, hospital level and drug level from 2017 to 2020 by nonproprietary name. In this study, market availability refers to the time lag in the approval of orphan drugs in China compare to international (the U.S., EU and Japan). Hospital-level availability is the percentage of orphan drugs that can be purchased in a particular institution, while drug-level availability is the percentage of hospitals in which a particular orphan drug can be purchased in all surveyed Hospitals. Using Wilcoxon rank-sum test to compare the differences of availability between years and areas. Differences in availability across years and regions were compared using the Wilcoxon rank-sum test.

The literatures indicated that the following criteria were used to describe the availability of orphan drugs [15, 16, 28]:

- Absent (0%): none of these orphan drugs were found in the surveyed institutions;
- Very low (< 30%): these orphan drugs were difficult to find in the surveyed institutions;
- Low (30–49%): these orphan drugs were not easy to find;
- Fairly high (50–80%): these orphan drugs were available at many of the surveyed institutions;
- High (80%): these orphan drugs were available in most institutions with good availability.

#### Affordability

According to the WHO Collaborative Center for Drug Statistics Methodology, we calculate the defined daily dose cost (DDDc) for each orphan drug by its DDD [24]. Differences in availability across years and regions were compared using the Wilcoxon rank-sum test.

$${\text{DDDc}}\;\left( {{\text{cost}}\;{\text{of}}\;{\text{defined}}\;{\text{daily}}\;{\text{dose}}} \right)\, = \,{\text{Median}}\;{\text{unit}}\;{\text{price}}\;{\text{of}}\;{\text{drug}}\;{\text{reported}}\;{\text{by}}\;{\text{institutions}}\, \times \,{\text{DDD}}\;({\text{defined}}\;{\text{daily}}\;{\text{dose}}).$$

The median price of unit dose was calculated based on the price per unit dose we collected from each institution (Additional file 6: Table S6 shows the median unit prices of generic orphan drugs surveyed in 30 leading provincial institutions in China from 2017–2020 (USD)).

Affordability was measured as the number of days a lowest paid unskilled government worker worked to pay for a treatment course for a particular drug based on the WHO/HAI methodology. We searched disposable daily income per capita of urban residents and net daily income per capita of rural residents published in the China Statistical Yearbook from 2017 to 2020 (Additional file 7: Table S7 shows the daily disposable income per capita of urban or rural residents from 2017 to 2020 in China) [29]. Because most rare diseases required lifelong medication, we measured affordability to compare the DDDc with residents' average daily income in this study. The National Basic Medical Insurance (NBMI) is the most comprehensive social medical insurance provided by the Chinese government, covering Part A and Part B drugs. Drugs included in Part A are free for people covered by NBMI, while 5–20% out-of-pocket expenses (OOPs) for Part B drugs. In addition, the price negotiation system in China was considered (Additional file 8: Table S8 shows the coverage of NBMI of surveyed orphan drugs in China from 2017–2020) [30].

#### Comprehensive analysis

We presented four quadrant diagrams by the drug-level availability and affordability in 2018 and 2020. According to the above availability and affordability criteria, drugs in quadrant IV have “fairly high” and “high” availability and affordable, which means these drugs have better availability and affordability.

## Results

### The national system for rare diseases in China

**Fig. 1:**
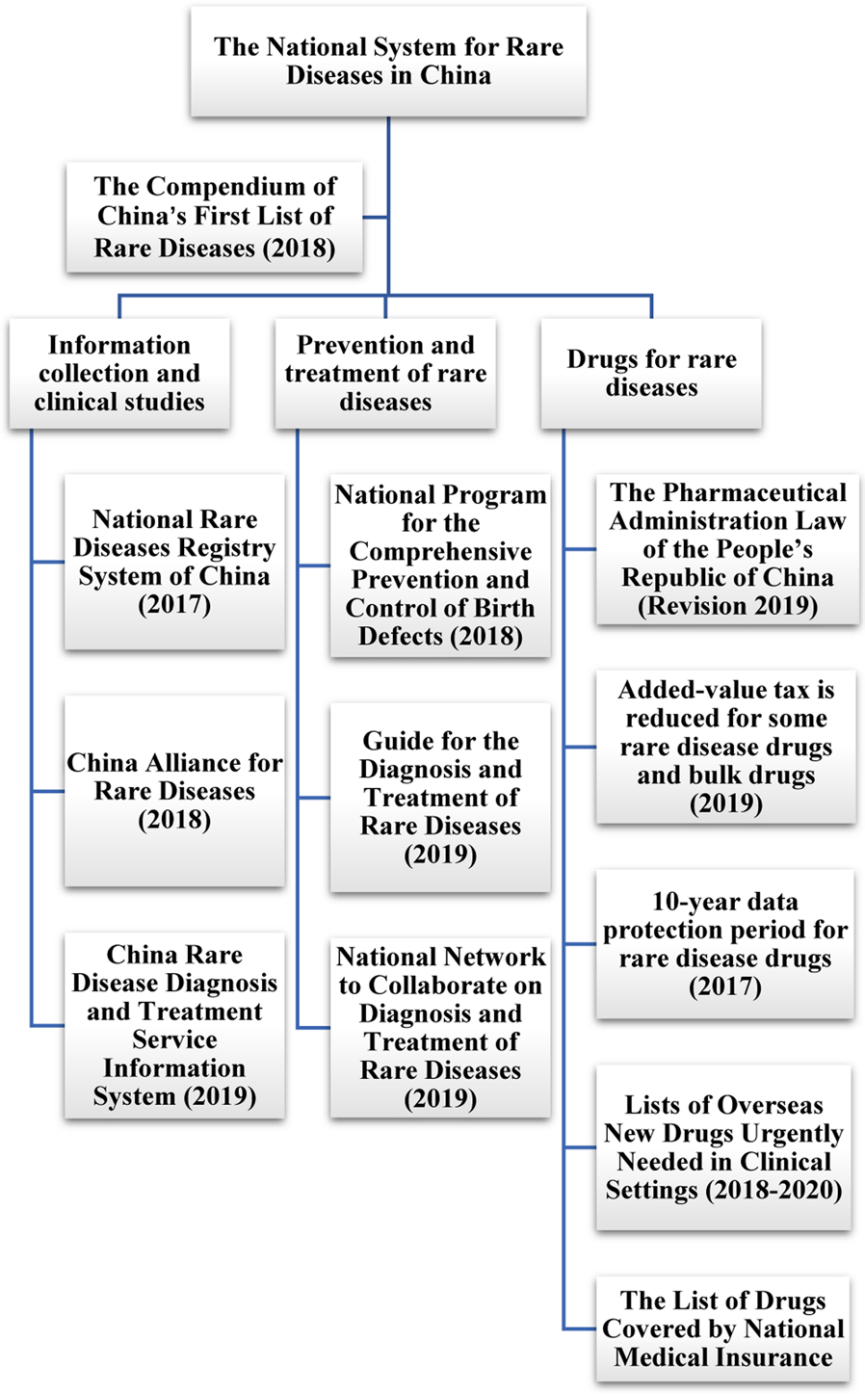
The national system for rare diseases in China. Based on the definition of rare diseases, the national system for rare diseases in China currently carries out from three aspects: information collection and clinical research, diagnosis and treatment, as well as drug policies, with each part has some crucial policies. Specifically, the Compendium of China’s First List of Rare Diseases (2018) (CLRD) has made China the first country to delineate the boundaries of rare diseases in the form of a catalogue, which is the core innovation of the system. Representative work in each aspect is listed in the figure

The national system of rare diseases in China currently carries out from three aspects: information collection and clinical research, diagnosis and treatment, as well as drug policies (Fig. 1). Specifically, the CLRD has made China the first country to define rare diseases in form of a catalogue, and it is an important foundation of the system that guides the development of other policies. The management of rare diseases is a complex and systematic work. It’s evident from the China’s national system that the rare diseases had received a high national attention and widespread social concern, especially in drug policies. From policy support to legal protection, China is gradually establishing a multi-party cooperation system for rare diseases and realizing resource integration, although it still needs continuous efforts to improve. The effectiveness of the system is urgently needed to be testified.

### Availability

#### Market availability

Among the surveyed drugs with CLRD definitions, the average time lag was 8.9 years (SD, 11.3) after the U.S., 2.1 years (SD, 10.9) after EU, and 5.3 years (SD, 12.9) after Japan until 2020. The number of surveyed orphan drugs approved from 2017 to 2020 is 20, exceeding the total number of orphan drugs approved in the 10-year period from 2007 to 2016 by 15. Among them, 27.8% of them were approved in 2017, 2018 and 2019 respectively and 16.7% were approved in 2020. In addition, according to ICD-11 [27], "endocrine, nutritional and metabolic diseases", "diseases of the nervous system" and "diseases of blood or hematopoietic organs" were in the top 3, accounting for 28.3%, 27.3% and 18.2%, respectively. Idiopathic pulmonary hypertension had the most approved drugs (9 drugs), followed by Parkinson's disease (young-onset, early-onset) and Multiple sclerosis, with 8 approved drugs each.

#### Hospital-level availability

Of total drugs, seven of them were not found in 30 institutions. The hospital-level availability of the remaining drugs nationwide and in 3 areas showed a significant upward trend from 2017 to 2020 (Table 1). By 2020, it reached 41.1% nationwide and 43.0% in eastern area. The increase of availability at hospital level in 2020 was significantly higher than that in 2018 (*P* = 0.01) and 2019 (*P* = 0.006), which meant that the availability in 2020 is significantly improved. In addition, hospital-level availability of drugs in all efficacy groups showed an increasing trend year by year. In particular, the median hospital-level availability of drugs for “developmental anomalies” was highest as of 2020, with 71.4% of drugs in this group available in surveyed institutions (Additional file 9: Table S9 Median of hospital-level availability for orphan drugs in each efficacy group). Proportion of imported and domestic drugs was 65.3% and 56.9% as of 2020, among which 22.2% had both imported and domestic drugs, domestic drugs accounted for more than half. In addition, majority of drugs were available in single dosage forms, seven drugs were available in two dosage forms, while Sirolimus is available in three dosage forms: tablet (imported), oral liquid and capsule (domestic) (Additional file 10: Table S10 shows the brand names and dosage forms of surveyed drugs in China.).

**Table 1:** Median availability of orphan drugs at hospital level in China from 2017 to 2020 (%)

Year (numbers of marketed orphan drugs)	Nationwide		Eastern area		Middle area		Western area		*p* value^c^
	Availability (IQR)^a^	Median of hospital-specific change (IQR)^b^	Availability (IQR)	Median of hospital-specific change (IQR)	Availability (IQR)	Median of hospital-specific change (IQR)	Availability (IQR)	Median of hospital-specific change (IQR)	
2017	27.2 (10.4)		31.0 (8.9)		24.7 (27.8)		27.2 (7.9)		< 0.001
2018	32.9 (10.1)	1.9 (5.1)**	34.8 (18.7)	1.9 (3.1)*	25.3 (32.3)	1.3 (4.7)	32.9 (10.1)	3.2 (6.6)*	< 0.001
2019	34.8 (11.4)	1.9 (3.5)	36.1 (19.3)	2.5 (5.4)	30.4 (29.1)	3.2 (2.8)	34.2 (10.1)	1.3 (4.7)	< 0.001
2020	41.1 (12.3)	5.1 (7.6)*	43.0 (20.3)	3.2 (10.8)	36.7 (27.8)	5.1 (9.2)	36.7 (14.6)	8.9 (9.2)*	< 0.001

^a^IQR: Interquartile range, equals to the difference between 75 and 25th percentiles

^b^Median of hospital-specific change is defined as the median of orphan drug availability annual change in each hospital. The *p* value of Wilcoxon rank-sum test for the difference of median of hospital-specifific change each year compared with the previous year: *for *p* < 0.05, **for *p* < 0.01

^c^*p* value of Wilcoxon rank-sum test for the difference of orphan drugs' average availability at hospital level between eastern, western, and middle areas each year

#### Drug-level availability

**Fig. 2:**
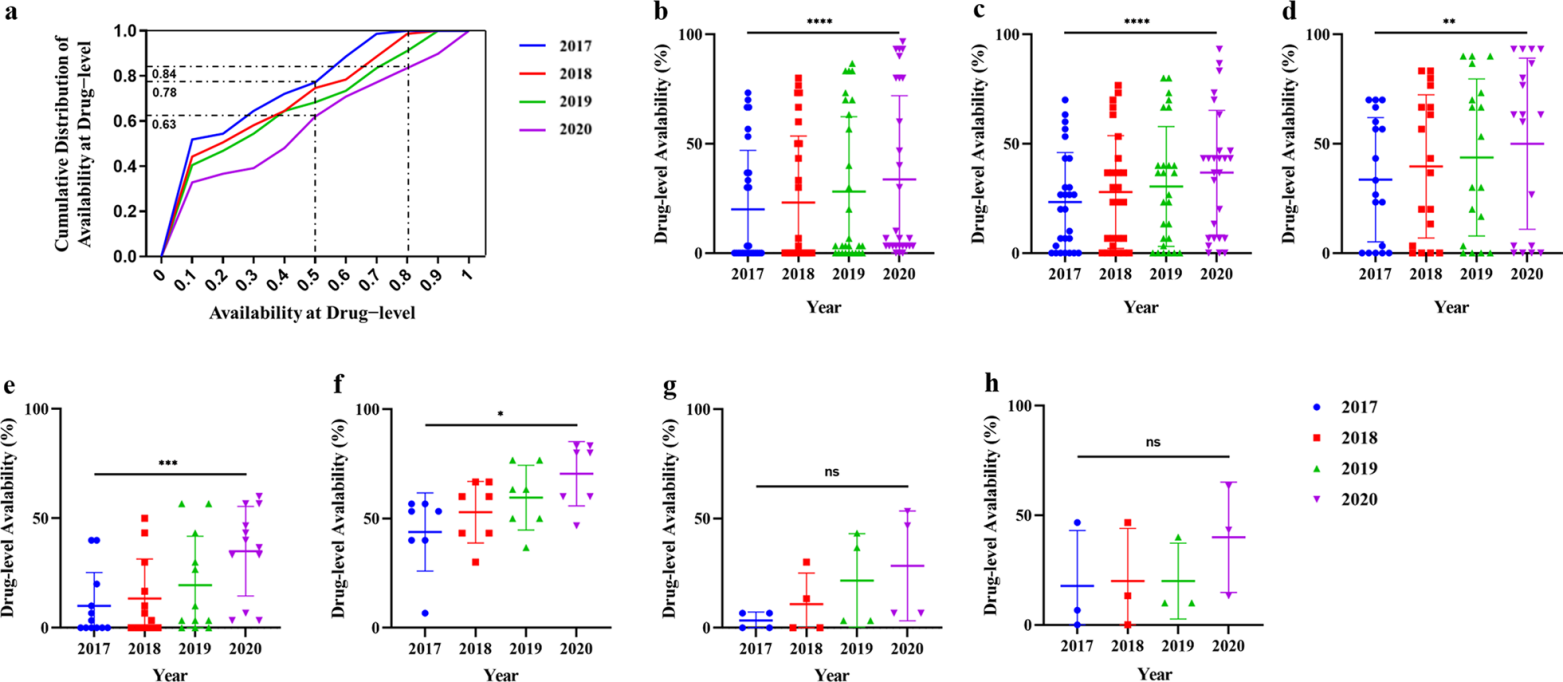
Availability of total drugs and efficacy groups at drug level from 2017 to 2020. The blue, red, green and purple represent the drug availability for 2017, 2018, 2019, and 2020, respectively. **a** Cumulative frequency distribution of total drug availability at drug level from 2017 to 2020. **b**–**h** Availability of 7 efficacy groups: **b** endocrine, nutritional and metabolic diseases. **c** Diseases of the nervous system. **d** Diseases of the blood or blood-forming organs. **e** Diseases of the circulatory system. **f** Developmental anomalies. **g** Diseases of the respiratory system. **h** Diseases of the immune system

Overall, the availability at drug level nationwide was 43.3% until 2020, with no significant difference among areas. The median availability in nationwide and in 3 areas also showed a growing trend from 2017 to 2020 (Additional file 11: Table S11 shows the median availability of orphan drugs at drug-level in China from 2017 to 2020 (%); Additional file 12: Table S12 shows the availability classification of orphan drugs at drug-level in China from 2017 to 2020 (%)). Cumulative frequency distribution of availability at drug level from 2017 to 2020 was shown in Fig. 2a. According to the literatures [15, 16, 28], about 77.2% and 62.1% of surveyed drugs were “low” availability, while 0.0% and 16.1% were at “high” level in 2017 and 2020, respectively. There was a huge leap in 2020 compared to 2017. Atorvastatin for Homozygous familial hyperlipidemia, which could be obtained in 29 hospitals, had the highest availability. Besides, we analyzed the drug-level availability of all efficacy groups. As shown in Fig. 2b–h, drug-level availability in all efficacy groups showed upward trends. As of 2020, median drug-level availability is highest for drugs to treat “developmental anomalies”. In contrast, median drug availability for drugs to treat “endocrine, nutritional and metabolic diseases” was the lowest at 6.67%, indicating that drugs for this efficacy group were available in 6.67% of surveyed institutions (Additional file 13: Table S13 Median of drug-level availability for orphan drugs in each efficacy group).

### Affordability

In order to ensure the accuracy of the survey, taking into account the different indications and dosage forms correspond to DDD values of the same drug, 99 drug-single indication matches were found in 30 hospitals. The median unit prices of drugs ranged from $0.00015/mg (Hydroxyurea) to $8574.8/mg (Nusinersen). By 2020, the median DDDc is $7.7, which is equivalent to 0.4 days' income for urban residents and 1.1 days' income for rural residents without NBMI considered. National DDDc showed a slight upward trend from 2017 to 2020 (Additional file 14: Table S14 shows the cost of DDD (DDDc) for surveyed orphan drugs from 2017 to 2020 in China (USD)).

In general, the range of the number of days a resident had to work under average daily income to pay for the DDDc of each drug was from 0.00004-day Tranexamic acid to 1052.9-day Coagulation factor VIIa. The maximum value of days decreased year by year, from 388.6 days in 2017 to 288.5 days in 2020 in urban areas, and from 1052.9 days in 2017 to 738.5 days in 2020 in rural areas. A total of 20 surveyed drugs were included in Part A, 3 of which were imported drugs. Besides, 27 drugs were included in Part B in 2018, 6 of which were imported drugs, while 42 drugs were included Part B in 2020, 18 of which were imported drugs. Among them, 7 drugs (Teriflunomide, etc.) were included into Part B after 2018 through price negotiation. Proportion of orphan drugs increased, and the reimbursement of imported drugs increased. We further analyzed the affordability of each efficacy group and total drugs under NBMI. As shown in Table 2, in both urban and rural areas, the affordability rates of all efficacy groups and total drugs showed a year-on-year increase. Under NBMI, the highest affordability rate was for orphan drugs that treated diseases of the nervous system, which reached 22.2% and 20.2% in urban and rural areas, respectively, in 2020; the most significant increase was for orphan drugs that treated “diseases of the circulatory system”, which increased by 7.1% and 5.1% in urban and rural areas, respectively. Overall, the affordability rate of rural residents rose from 49.5% in 2018 to 64.6% in 2020, and that of urban residents rose from 59.6% in 2018 to 74.7% in 2020 (Additional file 15: Table S15 shows the affordability for surveyed orphan drugs from 2018 to 2020 in China (USD)). It was found that NBMI significantly increased the affordability of orphan drugs, and reduced the urban–rural gap. By 2020, Alpha-galactosidase A for Fabry disease is the most unaffordable drug for residents, whose cost of one day's treatment is equivalent to 142.7 days' income of urban residents and 365.2 days' income of rural residents. This was followed by Imiglucerase for Gaucher's disease (124.9 days in urban, 319.7 days in rural) and Alglucosidase alfa for Glycogen storage disease (82.9 days in urban, 212.3 days in rural). All 3 drugs were not included in NBMI during the survey.

**Table 2:** Unit prices, DDDc ranges and affordability rate of surveyed drugs in each efficacy group in China from 2017 to 2020 (%)

ICD-11 categories of surveyed drugs	Median of unit prices (min–max)/(USD)^a^				Median of DDDc (min–max)/(USD)^b^				Urban			Rural		
									Rate of affordability^c^ (if 5% OOP^d^)			Rate of affordability (if 5% OOP)		
	2017	2018	2019	2020	2017	2018	2019	2020	2018	2019	2020	2018	2019	2020
Endocrine, nutritional and metabolic diseases	0.02 (0.00–579.02)	0.02 (0.00–563.04)	0.05 (0.00–563.04)	0.10 (0.00–506.17)	2.10 (0.06–57.32)	2.00 (0.07–56.42)	2.43 (0.09–1464.71)	10.77 (0.07–2519.71)	15.2	17.2	18.2	15.2	15.2	16.2
Diseases of the nervous system	0.04 (0.00–3.72)	0.03 (0.00–6.43)	0.03 (0.00–6.67)	0.07 (0.00–8574.75)	2.44 (0.02–53.31)	2.62 (0.03–53.31)	2.62 (0.03–30.95)	3.59 (0.04–857.48)	21.2	21.2	22.2	18.2	18.2	20.2
Diseases of the blood or blood-forming organs	0.14 (0.00–904.18)	0.14 (0.00–850.00)	0.14 (0.00–850)	0.14 (0.00–808.82)	10.68 (0.00–5696.30)	10.29 (0.00–5355.00)	10.24 (0.00–5355.00)	10.18 (0.00–5095.59)	11.1	11.1	11.1	9.1	9.1	9.1
Diseases of the circulatory system	0.20 (0.07–0.97)	0.25 (0.07–14.70)	0.73 (0.06–3516.62)	1.35 (0.06–3516.62)	15.15 (1.27–131.81)	22.40(1.27–146.96)	29.38 (1.27–146.96)	23.41 (1.27–131.77)	2.0	4.0	9.1	1.0	1.0	8.1
Developmental anomalies	5.16 (0.00–8.58)	5.00 (0.00–7.65)	4.86 (0.00–7.02)	4.66 (0.00–7.01)	7.30 (0.06–34.18)	6.84 (0.07–35.89)	6.53 (0.08–35.89)	6.52 (0.07–32.50)	7.1	8.1	8.1	3.0	5.1	5.1
Diseases of the respiratory system	2.02 (0.02–4.02)	2.12 (0.02–4.22)	0.37 (0.02–4.22)	0.23 (0.02–3.82)	38.24 (38.26–40.21)	39.24 (36.26–42.22)	76.01 (36.26–109.80)	38.24 (28.57–69.22)	1.0	2.0	3.0	1.0	2.0	3.0
Diseases of the immune system	0.04 (0.00–0.07)	0.04 (0.00–0.07)	0.07 (0.00–75.49)	0.06 (0.00–75.49)	0.36 (0.13–0.59)	0.37 (0.13–0.62)	0.59 (0.12–63.41)	0.59 (0.11–63.41)	2.0	2.0	3.0	2.0	2.0	3.0
Total	0.11 (0.00–904.18)	0.10 (0.00–850.00)	0.13 (0.00–3516.62)	0.20 (0.00–8574.75)	6.00 (0.00–5696.30)	6.43 (0.00–5355)	7.45 (0.00–5355)	10.68 (0.00–5095.59)	59.6	65.7	74.7	49.5	52.5	64.6

^a^Use the minimum specifications as the standards about the investigated drugs. Translated the price and took the median values as analysis objects

^b^In the calculations, we used the following average values: adult weight at 70 kg, children 15 kg, baby 1.5 kg; the body surface area at 1.7 m^2^

^c^Rate of affordability = Number of affordable drugs in each efficacy group/Number of drugs in each efficacy group × 100%. The 2017 NMBI implementation was mid-year, and to ensure accurate of data, we began analyzing affordability rates only from 2018

^d^“OOP”: out-of-pocket expenses

### Comprehensive analysis

**Fig. 3:**
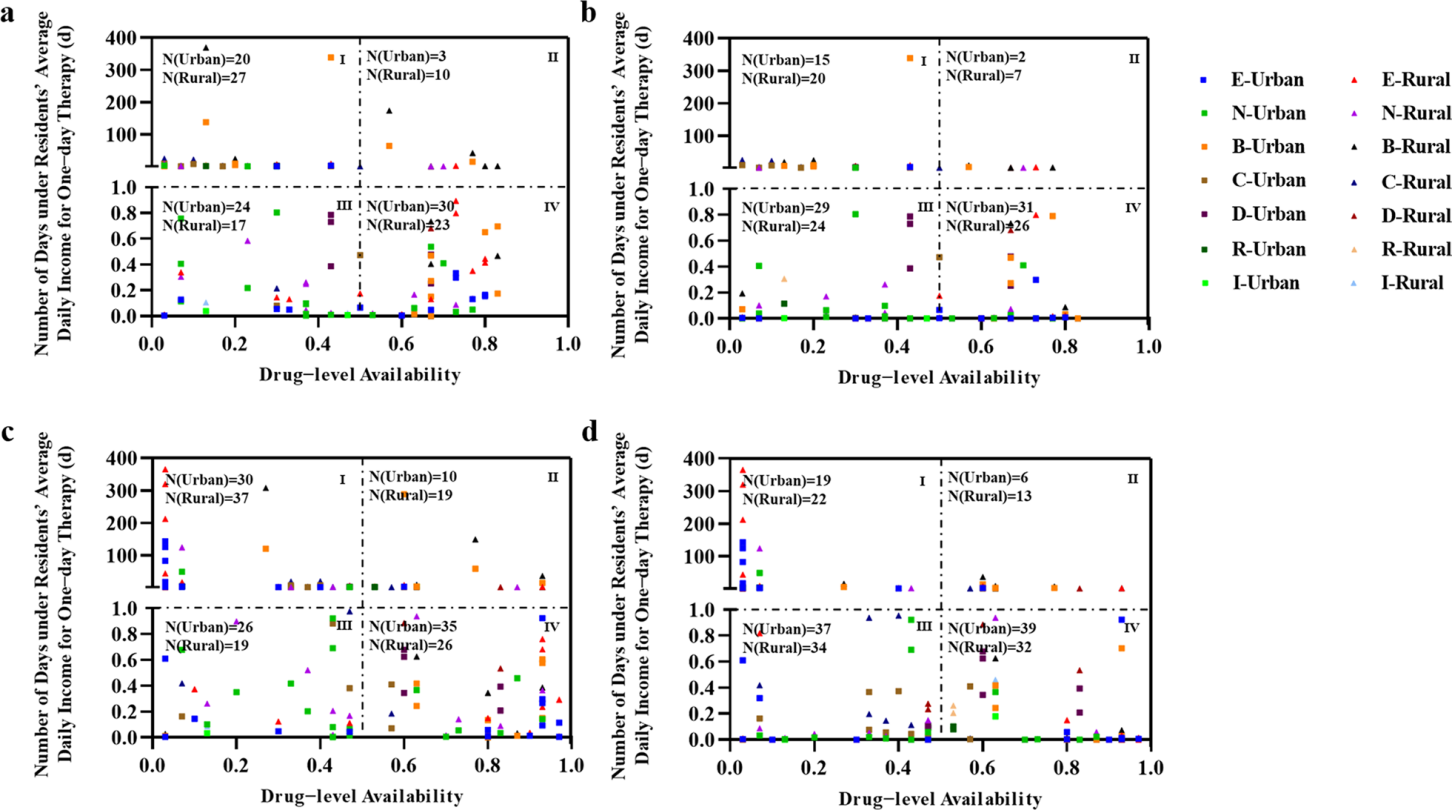
Four quadrant diagram of drug-level availability and affordability for drugs in different efficacy groups for urban and rural residents. **a** Drug-level availability and affordability without NBMI for urban and rural residents in 2018. **b** Drug-level availability and affordability with NBMI for urban and rural residents in 2018. **c** Drug-level availability and affordability without NBMI for urban and rural residents in 2020. **d** Drug-level availability and affordability with NBMI for urban and rural residents in 2020. E: Endocrine, nutritional and metabolic diseases; N: Diseases of the nervous system; B: Diseases of the blood or blood-forming organs; C: Diseases of the circulatory system; D: Developmental anomalies; R: Diseases of the respiratory system; I: Diseases of the immune system

We presented four quadrant diagrams by the drug-level availability and affordability in 2018 and 2020 without or with NBMI considered (Fig. 3). Under NBMI, the highest number of drugs with better availability and affordability was for orphan drugs that treated “endocrine, nutritional and metabolic diseases”, which were 11.1% and 10.1% in urban and rural areas, respectively, in 2020. The number of drugs with better availability and affordability that treated “developmental anomalies”, “diseases of the circulatory system”, and “diseases of the nervous system” were growing from 2018 to 2020 in both urban and rural areas. In general, the number of total drugs with better availability and affordability in quadrant IV increased among urban and rural residents (Additional file 16: Table S16 Number of orphan drugs with better availability and affordability in each efficacy group). The orphan drugs with better availability and affordability were 39 in urban areas and 32 in rural areas by 2020, respectively, which was still at a low level although rising.

## Discussion

In this study, we conducted an annual and regional comparative analysis of access to orphan drugs in China, hoping to provide a reference for the development of countries with a late start in rare diseases in the world by taking the national system in China as an example. There were some studies have assessed the accessibility of orphan drugs in China in past few years [15, 16]. In comparison, this study has some remarkable features. First, CLRD is the first definition of rare diseases in China, and our study delineates the range of diseases with CLRD, which is the first study based on CLRD since its release in 2018, and is more accurate and comprehensive compared with previous studies. Secondly, NCDTRD is the nationally designated institutions for rare disease diagnosis and treatment in each region. PUMCH is the only leading national institution, and there are 32 provincial lead institutions, 30 of which are included in our survey. This is the first study to use provincial lead institutions as data sources, which made the data collected more reliable. Thirdly, our study includes cross-sectional comparisons for three economic disparities and longitudinal comparisons for four years from 2017 to 2020. In addition, the availability and affordability analyses for seven efficacy groups were analyzed, making it the most comprehensive analysis of rare disease drug accessibility in China. Finally, the background and time span of this study was selected around the establishment of the National System for Rare Diseases in China, with the aim of analyzing the effectiveness of the system from the perspective of orphan drug accessibility, which has a more ambitious meaning.

Despite differences in research, all related studies came to similar conclusions: access to drugs for rare diseases in China was still low, but has shown a clear upward trend in recent years. This corroborates the conclusion of this study that the national system of rare diseases in China has achieved significant results in terms of drug access. Specifically, there are four main findings in this study. First, the market availability of orphan drugs in China is still relatively low in 2020. The number of drugs approved between 2017 and 2020 exceeds the number approved in the decade 2007 to 2016, indicating that the implementation of the China’s national system for rare diseases played an important role in the increasement of market availability. Meanwhile, there are thirteen orphan drugs for ten rare diseases of the CLRD were approved in China in 2021, making a huge leap in access to orphan drugs. In addition, it should also be noted that compared with other three countries, the marketing time of surveyed drugs still lagged behind, which is consistent with previous findings, indicating that the R&D of orphan drugs is still the top priority of rare disease management in China. The independent R&D capability of rare disease drugs is the main factor affecting the price of rare disease drugs, and it is also the key to restricting the accessibility of the Chinese market. It is closely related to policy management. The development of rare disease management in China lags behind that of European and the U.S., and this leaves rare disease definition and management policies still to be improved. Definition of rare diseases, which is the cornerstone of R&D, has not been fundamentally resolved for a long time in China [31]. Considering national conditions, the definition will coexist with the updated list for a period of time to come. Although this way of defining is an adaptive policy in the early stages of rare disease development, our findings suggest that it has yielded optimistic results, at least in terms of drug accessibility. In addition, national policy incentives are the driving force behind independent R&D. There are two barriers to R&D of orphan drugs: first, the number of patients with rare diseases is fairly small, and many diseases have not yet developed accurate diagnosis and treatment level; second, return on investment is an issue that drug institutions must consider, while orphan drugs rely on more advanced instruments, which means greater investment and risks [32]. In the US, orphan drug clinical research costs can be tax-deductible by 50%, with extended tax-deductible periods and tax relief for orphan drugs. Exempting applicants for orphan drugs from FDA review fees, and a 7-year market monopoly period. In China, it’s better to direct the incentives toward orphan drugs with low availability, and more specific incentives are needed to establish targeted subsidies for orphan drugs. For imported orphan drugs, further promote the use of real-world research, an orphan drug import tariff reduction system and a free public platform for all stakeholders to share information on orphan drugs also need to be developed. International cooperation will play a crucial role in the response to rare diseases [33]. Besides, the high market accessibility rare diseases (endocrine, nutritional and metabolic diseases, etc.) reflects the morbidity and treatment needs of rare diseases in China (oncology and infectious diseases are not included in the CLRD). Policies should be guided by market demand. There is a certain information block between patients with rare diseases and drug development institutions, and patient organizations can play an important role in this process. The National Organization for Rare Disorders in the US, EURODIS in Europe and Canadian Organization for Rare Disorders have played an vital role in leading the development of orphan drug legislation, supporting patients, raising awareness and sponsoring academic research [1, 34–36].The number of patient organizations in China has grown rapidly in recent years. However, these organizations are lack of training, play no role of promoting legislative agenda, and generally not active in the academic research. It’s hoped that with the implementation of government policies, the awareness of rare diseases can be better deepened in China.

Second, the availability of surveyed drugs increased year by year in three areas and in nationwide. In 2020, the hospital-level availability increased to more than 1.5 times that of 2017, with domestic drugs accounting for more than half, indicating that the China’s national system has achieved significant results. However, seven drugs were absent in the surveyed institutions, five of them were approved 5 years before 2020. Drugs for “endocrine, nutritional and metabolic diseases” and “diseases of the nervous system”, the two efficacy groups with the highest market availability, had the lowest hospital-level availability. This indicated a significant delay of hospital orphan drugs access. The reasons might be related to the low ability of diagnosis and treatment, the immature procurement and management system of orphan drugs. The establishment of the collaboration network is a good demonstration, but the specific resource scheduling of the huge collaboration network still needs to be considered. Doctors' professionalism of rare diseases also needs to be enhanced, especially in primary institutions [37, 38]. At present, online multi-disciplinary team for rare diseases with primary institutions is a referable approach, but it’s not a fundamental solution [10]. In addition, there is no clear orphan drugs procurement policies and use of norms, and no special management measures in the hospital pharmacy in China. A study to assess the accessibility of explicit diagnosis of rare diseases in Chinese adults showed that about 72.97% of patients were misdiagnosed, and patients waited an average of 4.30 times and visited 2.97 hospitals before being diagnosed [39]. In the future, more attention should be paid to the uneven distribution of medical networks for rare diseases and quality medical services. The drug ratio and the government's control over the total amount of medical insurance are important assessment indicators for public hospitals, which need to control the drug ratio at around 30% overall. The procurement of drugs for rare diseases, which cost more but have "less clinical demand", undoubtedly poses a greater challenge for hospital pharmacy management. It's all about drug prices. In addition, the availability at drug level also showed an increasing trend by year and by region, which also confirmed the remarkable effect of the system. However, it should also be noted that the proportion of “high” availability drugs remained low (16.5%), and the availability of high-price drugs was low. By 2020, Alpha-galactosidase A, Imiglucerase and Alglucosidase alfa, the top three most unaffordable orphan drugs for residents under NBMI, were all in the "very low" grade of availability (3.3%). It suggested that improving the availability of high-priced drugs, whether through imports or independent R&D, was the focus of future efforts. Besides, drugs for “endocrine, nutritional and metabolic diseases” and “diseases of the nervous system” also had the lowest drug-level availability. The future policies should be more prioritized towards these two efficacy groups.

Thirdly, the median DDDc increased by year during 2017–2020. It has been reported that the median DDDc of orphan drugs in China showed a decreasing trend from 2010 to 2017, which is different from our finding [15]. This may be related to the newly approved high-price orphan drugs encouraged by rare disease policies in recent years. Our results show that the average DDDc of newly approved drugs in the four years is $54.0, $56.4, $865.3 and $35.6, respectively. Moreover, these are beyond the affordable range of urban residents in 2020 (equivalent to 2.0–49.0 days of urban residents' income from work) and rural residents' affordable range (equivalent to 5.2–125.4 days of rural residents' income from work), which also confirms our conclusions above. The underlying problem behind this is that patients are not able to take the full course of drugs. According to the Chinese Organization for Rare Disorders, 58% of registered rare disease patients received treatment, but most of them failed to take adequate drugs. It has been shown that the use of high-cost drugs impoverishes most families [40, 41]. Future rare disease incentives should be pursued simultaneously in terms of both improving availability and reducing the price of high-priced drugs.

Finally, the economic burden of patients with rare diseases is decreasing, but the overall affordability is still not optimistic. By 2020, the affordability rate was 64.6% in rural areas and 74.7% in urban areas, respectively, but the proportion of better availability and affordability drugs was still not unfavorable (39.4% in urban areas after NBMI), this reflected a mismatch between the affordability and accessibility of some affordable drugs. This once again reflects that the provincial and hospital drug procurement and the development of national medical insurance have not been synchronized. Among them, problems such as prescription restrictions, procurement restrictions and the disadvantages of designated hospitals remain to be resolved. The list of Part A and Part B includes 62.6% of the surveyed drugs. The proportion of surveyed orphan drugs included in Part B increased from 27.3% to 42.4% during our survey, and the proportion of imported drugs also increased significantly, further explaining that the NBMI has attached more importance to rare disease drugs. Teriflunomide and other 7 drugs have been included in Part B reimbursement through price negotiation after 2018. After being reimbursed, all 7 drugs have changed from unaffordable to affordable. It further indicated that the price negotiation system and NBMI play an important role in the affordability of orphan drugs, and significantly narrow the affordability gap between urban and rural areas. China has a breakthrough in the guarantee of high-price orphan drugs as of 2020. Nusinersen for spinal muscular atrophy and Alglucosidase alfa for Fabre disease have been included in the National Catalogue of Medical Insurance [42]. In addition, since 2005, Shanghai, Qingdao, Zhejiang and other places have introduced rare disease drug coverage models with local characteristics, such as Qingdao's supplementary medical insurance system, which has helped patients alleviate payment problems to a certain extent. However, there’s still a lot of room for the improvement in China's payment system for orphan drugs. Multi-criteria decision analysis has recently been considered as a proper tool for orphan drug payment research [43–46]. International experience shows that multi-party healthcare payment is the ideal model for orphan drugs, and China should consider developing matching payment schemes, such as installment payment and efficacy insurance [47]. In addition, there have been a number of donated drug projects in China, including hemophilia, pulmonary hypertension, multiple sclerosis drugs and so on. These measures will have a meaningful impact on affordability. There is already a wealth of international experience in this area. Defining rare diseases through legislation, encouraging a differentiated registration and approval system for orphan drugs, building a government-led, health insurance-covered, multi-payer drug protection system, and setting up a differentiated health technology assessment system, while reducing or even eliminating out-of-pocket costs for patients, are the mainstream models at present [48–51]. China's orphan drug management system should learn from its reference.

## Conclusions

Although there is still a gap compared to countries with early development of rare diseases, the accessibility of orphan drugs in China improved by year from 2017 to 2020, which preliminary verified that the national system for rare diseases in China has made significant achievements in terms of orphan drug access. This study maps the accessibility of orphan drugs in China. In the future, China will continue to update the CLRD and strengthen the cooperation among the institutions of the national network, thus gradually improving national system for rare diseases.

## Supplementary Information

**Additional file 1: Table S1.** The questionnaire on the availability and prices of surveyed orphan drugs in leading provincial institutions.

**Additional file 2: Table S2.** List of 79 orphan drugs surveyed in 30 public tertiary hospitals in China (99 drug-indication matches).

**Additional file 3: Figure S3.** The flow chart for orphan drug screening in the survey.

**Additional file 4: Table S4.** ICD-11 categories of surveyed drugs in China.

**Additional file 5: Table S5.** List of the surveyed 30 leading provincial institutions of the National Network to Collaborate on Diagnosis and Treatment of Rare Diseases in China.

**Additional file 6: Table S6.** The median unit prices of generic orphan drugs surveyed in 30 leading provincial institutions in China from 2017-2020 (USD).

**Additional file 7: Table S7.** The daily disposable income per capita of urban or rural residents from 2017 to 2020 in China.

**Additional file 8: Table S8.** The coverage of NBMI of surveyed orphan drugs in China from 2017-2020.

**Additional file 9: Table S9.** Median of hospital-level availability for orphan drugs in each efficacy group.

**Additional file 10: Table S10.** Brand names and dosage forms of surveyed drugs in China.

**Additional file 11: Table S11.** Median Availability of Orphan Drugs at Drug-Level in China From 2017 to 2020 (%).

**Additional file 12: Table S12.** Availability classification of Orphan Drugs at Drug-Level in China From 2017 to 2020 (%).

**Additional file 13: Table S13.** Median of drug-level availability for orphan drugs in each efficacy group.

**Additional file 14: Table S14.** The cost of DDD (DDDc) for surveyed orphan drugs from 2017 to 2020 in China (USD).

**Additional file 15: Table S15.** The affordability for surveyed orphan drugs from 2018 to 2020 in China.

**Additional file 16: Table S16.** Number of orphan drugs with better availability and affordability in each efficacy group.

## Data Availability

The datasets generated and/or analysed during the current study are not publicly available due to the detailed data involving non-public drug information of surveyed institutions but are available from the corresponding author on reasonable request.

## Abbreviations

FDA: Food and Drug Administration
CLRD: Compendium of China’s First List of Rare Diseases (2018)
NCDTRD: National Network to Collaborate on Diagnosis and Treatment of Rare Diseases
ICD-11: International Classification of Diseases 11th Revision
HAI: Health Action International
DDD: Daily defined dose
DDDc: Defined daily dose cost
NBMI: National Basic Medical Insurance
ATC: Anatomical therapeutic chemical
OOPs: Out-of-pocket expenses
